# Recent Advances in the Fabrication of High-Performance Polypropylene Micro-Nano Composites via Supercritical Foaming

**DOI:** 10.3390/ma19081527

**Published:** 2026-04-10

**Authors:** Xin Pan, Gang Wang, Faqi Zhan, Yuehong Zheng, Mengyao Dong, Peiqing La, Kun Li, Xiaoli Zhang, Jingbo Chen

**Affiliations:** 1 State Key Laboratory of Advanced Processing and Recycling of Non-Ferrous Metals, School of Materials Science and Engineering, Lanzhou University of Technology, Lanzhou 730050, Chinazhanfaqi@lut.edu.cn (F.Z.); zyh@lut.edu.cn (Y.Z.); 2 Key Laboratory of Material Processing and Mold Technology, School of Electrical Engineering, Chongqing Industry Polytechnic University, Chongqing 401120, China; wanggang@cqipc.edu.cn (G.W.);; 3 School of Materials Science and Engineering, Zhengzhou University, Zhengzhou 450001, China

**Keywords:** supercritical foaming, polypropylene micro-nano composites, performance regulation, fabrication process, applications

## Abstract

Against the backdrop of the global trends toward lightweighting, multi-functionalization, and greening of materials, polypropylene (PP) has been extensively applied owing to its advantages of low density and low cost. However, its inferior foaming performance fails to meet high-end application requirements, which is primarily attributed to its low melt strength and restricted crystallization behavior. In this paper, the five-dimensional selection mechanism and classification of components for PP micro/nanocomposites fabricated via supercritical foaming are systematically summarized. The regulatory effects of micro/nano additives on the crystallization, rheological properties, and foaming behavior of PP are quantitatively analyzed. The parameter optimization windows of three foaming processes, namely batch foaming, extrusion foaming, and injection foaming, are integrated (e.g., a foaming temperature of 150–170 °C and a saturation pressure of 8–20 MPa). Additionally, the application progress of PP micro/nanocomposite foams in fields such as automotive lightweighting (with a weight reduction rate of 64.29%) and building thermal insulation (with a thermal conductivity as low as 29 mW/(m·K)) is outlined. The core novel insight of this work lies in clarifying the unified mechanism of crystal refinement induced by reinforcing agents with different geometric morphologies, which is dominated by the synergy between heterogeneous nucleation and steric hindrance. This finding provides theoretical and technical guidelines for the industrial-scale preparation of high-performance PP foams.

## 1. Introduction

Against the backdrop of the global materials industry transitioning towards lightweighting, multi-functionality, and greenization [1,2,3], general thermoplastic polymers have become core materials in automotive, construction, electronic information, and other fields due to their excellent processing adaptability and cost advantages [4,5]. As one of the highest-yield general polyolefins, polypropylene (PP) has been widely applied owing to its low density (0.90–0.91 g/cm^3^) [6], outstanding chemical stability [7], high processing fluidity [8], and low cost [9].

However, the linear molecular chain structure of PP leads to low melt strength and poor viscoelasticity, resulting in defects such as cell collapse and coalescence during foaming. This makes the performance of pure PP foams unable to meet the requirements of high-end applications. For instance, the cell diameter of pure isotactic polypropylene (iPP) foams can reach 174.63 μm, with a cell density of only 5.07 × 10^5^ cells/cm^3^ and a thermal conductivity as high as 40–50 mW/(m·K) [10]. The 5G base station casings require electromagnetic interference shielding effectiveness (EMI SE) > 5 dB [11] and lithium battery thermal insulation pads thermal conductivity < 30 mW/(m·K) [12]. Additionally, PP exhibits a slow crystallization rate and its crystal structure is susceptible to processing conditions, which further limits the optimization space of its foaming performance [13,14,15]. Thus, modification methods are urgently needed to break through the aforementioned bottlenecks.

To enhance the performance and functional integration of PP foams, researchers have adopted chemical modification and physical modification [16]. Among them, micro-nano composite modification is the current research hotspot. The micro or nano fillers were introduced into polypropylene matrix to achieve the synergistic reinforcement of composite properties [17]. It also solves the problems of insufficient melt strength and low nucleation efficiency [18]. Supercritical foaming technology, using supercritical CO_2_(scCO_2_) or N_2_(scN_2_) as physical blowing agents, is a core solution for PP foaming pain points, featuring environmental friendliness, high gas solubility, and controllable cell structure [19]. Compared with traditional chemical/solvent-based physical foaming, it enables directional cell structure design of PP micro-nano composites via precise control of saturation pressure and foaming temperature [20]. Additionally, the scCO_2_ plasticizing effect reduces PP crystallization temperature and accelerates crystallization, synergizing with micro-nano reinforcements’ heterogeneous nucleation to optimize foam performance [21]. This technology has developed into batch foaming (laboratory precision regulation) [22], continuous extrusion foaming (large-scale production) [23], and precision open-mold injection foaming (complex part fabrication) [24].

Based on this, this paper systematically reviews the research progress in the preparation of PP micro-nano composites via supercritical foaming. It first elaborates on the composition design and compounding mechanism of PP micro-nano composite systems. Then, it explores the regulatory mechanisms of micro-nano reinforcements on the crystallization behavior, rheological properties of PP, and their synergistic effects with the supercritical foaming process. The integration of supercritical foaming processes (batch, extrusion, injection) and parameter optimization rules are analyzed. Subsequently, the application status of such materials in automotive lightweighting, building fireproof insulation, packaging protection, and emerging fields is summarized. Finally, the key bottlenecks in current research are pointed out, and the future development directions of raw material innovation, process intelligence, and functional integration are prospected. This paper provides relevant enlightenment and reference or guidance for researchers.

## 2. Selection Mechanism and Classification of Micro-Nano Additives

### 2.1. Properties of PP Matrix

As a typical semi-crystalline thermoplastic, PP possesses intrinsic physicochemical properties that make it a preferred matrix for high-performance supercritical fluid foamed materials. Its linear molecular chain with saturated C-C bonds and methyl side groups endows excellent resistance to acids, alkalis, organic solvents, and supercritical fluids, while supercritical CO_2_ solubility in PP lays the foundation for fine, dense cell formation [25]. PP exhibits superior lightweight mechanical synergy: its intrinsic density (0.90–0.91 g/cm^3^) drops to 0.1–0.5 g/cm^3^ post-foaming, and crystallinity adjustment balances rigidity and toughness, resulting in >90% closed cell content and mechanical stability exceeding expanded polystyrene and polyethylene [26]. The three main types of PP foam materials are shown in Table 1.

With a suitable melt flow index, PP is compatible with conventional injection molding/extrusion equipment, reducing industrialization costs [27,28]. Economically and environmentally, PP can be recycled and paired with non-toxic supercritical foaming, aligning with sustainability trends [29]. Despite these advantages, PP’s inherent properties and crystallization characteristics induce three critical bottlenecks limiting foam quality and industrialization. First, linear molecular chains with insufficient entanglement lead to low melt strength, causing cell wall rupture/coalescence during expansion [30,31]. Long-chain branching modification improves melt strength but increases process complexity and costs by 10–30%. Second, sensitive crystallization kinetics results in poor foam uniformity, with cell distribution coefficient of variation > 20% due to uneven industrial cooling [32,33]. Third, dominant α-crystal structure and high low-temperature crystallinity cause poor toughness: impact strength decreases by 30–50% below 0 °C, and toughening via crystallinity reduction sacrifices rigidity [34]. To address these intrinsic bottlenecks, micro-nano additives have emerged as a versatile modification strategy, simultaneously regulating the matrix structure and the foaming mechanisms.

**Table 1:** The three main types of PP foam materials.

PP Type	Molecular Structure	Crystallinity	Cell Density (Cells/cm^3^)	Ref.
iPP	Linear chain	65%	5.07 × 10^5^~2.3 × 10^9^	[18,35]
LCBPP	Long-chain branched	55%	1.0 × 10^10^~5.4 × 10^11^	[36]
HPP	Blended elastomer	45%	8.0 × 10^8^~3.5 × 10^9^	[37]

### 2.2. Selection Mechanism of Micro-Nano Modification Reinforcements

The selection of micro-nano additives in the PP matrix for supercritical foaming relies on five core mechanisms, which synergistically address PP foaming bottlenecks and ensure the stability of the “polymer–gas” homogeneous system.

(1) Thermal stability. The thermal decomposition temperature of micro-nano additives must exceed the supercritical CO_2_ foaming temperature of PP [38]. This prevents additive degradation during foaming, thereby maintaining the thermal stability of the foaming system. (2) Compatibility. Additives should form stable interfacial interactions (e.g., van der Waals forces, hydrogen bonds, and chemical bonds) with PP molecular chains to optimize interfacial bonding and mitigate phase separation [39,40], effectively suppressing gas escape during foaming. (3) CO_2_ solubility promotion. Additives need to possess interaction sites (e.g., dipole interactions or van der Waals forces) with CO_2_, which enhances CO_2_ solubility in the PP matrix [41]. This mechanism lays the foundation for constructing the homogeneous “polymer–gas” system required for supercritical foaming. (4) Melt strength enhancement. The melt strength and elasticity of PP are improved via two pathways: (i) introducing long branches on PP molecular chains to increase chain entanglement density [42]; and (ii) forming a network structure in the matrix (e.g., graphene) to restrict molecular chain sliding [43], in order to inhibit cell wall rupture during foaming process. (5) Nucleation assistance. Micro-nano additives act as heterogeneous nucleation sites, reducing the nucleation free energy barrier (by >70% compared to homogeneous nucleation) to improve nucleation efficiency and cell uniformity [44]. This mechanism supports the preparation of microcellular (<10 μm) and nanoporous PP foams.

This correlation fills the adaptation gap between PP and supercritical foaming, providing core support for subsequent process optimization, performance regulation, and application expansion.

### 2.3. Classification of Micro-Nano Reinforcements

With the design objectives of adapting to supercritical foaming processes, synergistically regulating foam properties, and endowing functional characteristics, the core components of PP micro-nano composites form a synergistic system through precise proportioning [45,46].

**Figure 1:**
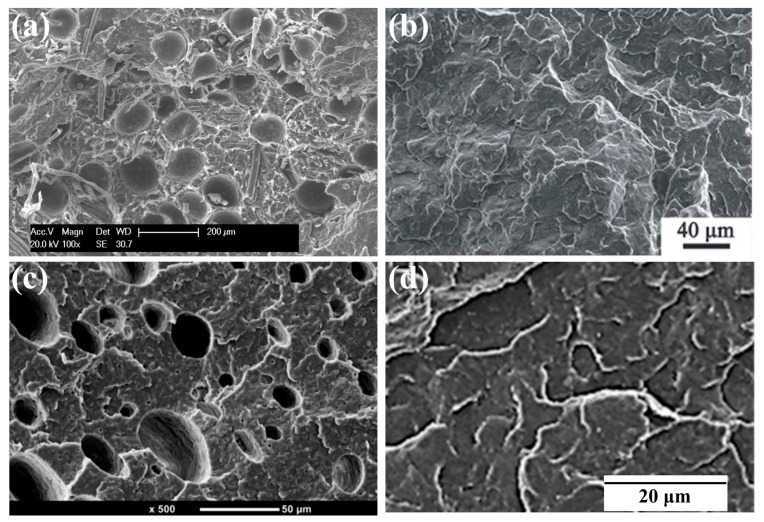
Dispersion morphologies of different reinforcements: (**a**) PP/CFA (1 wt.%) [55]; (**b**) PP/MS (2 wt.%) [56]; (**c**) PP/PTFE (1 wt.%) [57]; (**d**) PP/POE (50 wt.%) [54].

Specifically, micro-nano reinforcements are classified into four categories based on their materials. (1) Natural fibers (bamboo fiber, BF; sisal fiber, SFS) with a content range of 10–40%, where 20 wt.% BF can enhance the tensile strength of PP foam by 57% and under the testing conditions of foam density of 0.1 g/cm^3^ and compression rate of 1 mm/min, the incorporation of 40 wt.% SFS enhances the compressive strength of PP foam from 0.05 MPa to 1.55 MPa [16,47] (Figure 1a). (2) Inorganic nanoparticles (nano-TiO_2_, carbon nanotubes, CNTs, graphene, and MS) with a content of 1–15%; for instance, 3 wt.% nano-TiO_2_ reduces the spherulite diameter of PP from 80 μm to 20 μm and increases the crystallization temperature by 3 °C, while 12 wt.% CNTs enable the electromagnetic interference shielding effectiveness (EMI SE) of iPP/HDPE bilayer foam to reach 37.32 dB [48,49,50] (Figure 1b). (3) Polymer microfibers (polybutylene terephthalate, PBT; polytetrafluoroethylene, PTFE microfibers) with a content of 2–8%, among which 8 wt.% PBT microfibers (aspect ratio > 100) increase the melt complex viscosity (η) by three times, and 5 wt.% PTFE microfibers inhibit cell coalescence and reduce the cell diameter by one order of magnitude [51,52] (Figure 1c). (4) Elastomers (ethylene-propylene-diene monomer, EPDM; polyolefin elastomer, POE) with a content range of 5–20%, where 10 wt.% EPDM increases the impact toughness of BF/PP foam by 34.42% and 20 wt.% POE allows the open cell content of PP foam to exceed 80% [53,54] (Figure 1d).

## 3. Molding Processes and Optimization

### 3.1. Types of Supercritical Foaming Molding Processes

#### 3.1.1. Batch Foaming

**Figure 2:**
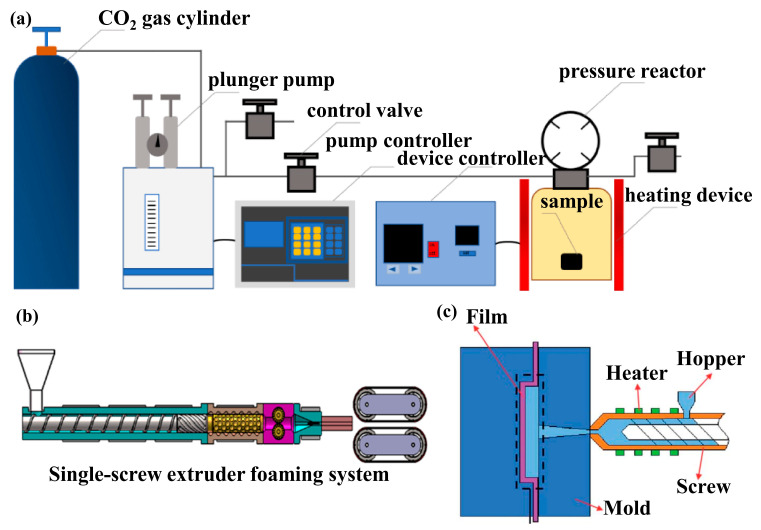
Schematic diagrams of different supercritical foaming processes: (**a**) batch foaming; (**b**) extrusion foaming [62]; (**c**) injection foaming [63].

Depending on the differences in production scale and product requirements, the specific processes and technical key points of supercritical foaming vary significantly. Batch supercritical foaming is mainly applied in laboratory research and small-batch production, with three core stages: (1) melting–saturation: dissolution of _SC_CO_2_ in PP melt; (2) crystallization induction: cooling to annealing temperature to form partially melted crystals; (3) rapid depressurization and cooling to finalize the structure. Its core advantage is precise cellular structure regulation. Thermodynamic supersaturation (triggered by rapid depressurization or heating) initiates nucleation, followed by gas dissolution–diffusion for cell growth and rapid cooling for stabilization. It features high nucleation efficiency, uniform cell morphology, and high cell density (Figure 2a).

Nucleation efficiency (*η*_n_) is defined as the ratio of the actual nucleation number to the theoretical maximum nucleation number, which is calculated by the formula:

(1) $$\eta_{n}=\frac{N_{a}}{N_{t}}$$

where $N_{a}$ denotes the actual nucleation number with the unit of cells/cm^3^, and $N_{t}$ represents the theoretical maximum nucleation number derived from the classical nucleation theory. Specifically, $N_{t}$ is expressed as [58]:

(2) $$N_{t}=\frac{{\left(∆G_{nucleus}\right)}^{2}}{kTln(S)}$$

In the aforementioned formulas, $∆G_{nucleus}$ is the nucleation free energy, k is the Boltzmann constant, T refers to the foaming temperature, and S stands for the supersaturation degree.

#### 3.1.2. Extrusion Foaming

Continuous extrusion foaming focuses on large-scale production, with a process that sequentially includes raw material mixing (blending PP, reinforcements, and additives in proportion), melt blending, supercritical gas injection, homogeneous system formation (achieving uniform component dispersion through shear action in the mixing section), die foaming (controlling the temperature at 150–170 °C and realizing a rapid pressure drop), and cooling setting [24]. The technical keys of this process are screw structure design and die temperature control—for example, the use of a barrier screw can significantly improve mixing efficiency, and by optimizing process parameters, PP/POE blends can be processed via continuous extrusion foaming to prepare oil-absorbing foams with high open-cell content (85%) [59] (Figure 2b).

#### 3.1.3. Injection Foaming

Precision open-mold injection foaming integrates rapid heat cycle molding and precision open-mold technology, with a specific process: First, the melt injection is performed, followed by gas saturation with scN_2_. Then, the mold is heated to 120 °C via rapid thermal cycling. Subsequently, precision open-molding is completed with an opening distance of 2–10 mm and an opening rate of 50 mm/s. Finally, the finished product is obtained after cooling setting [60]. Sandwich-structured foams were prepared using environmentally friendly scN_2_ as the physical blowing agent, with the fabrication process and final morphology shown in Figure 2c [61]. The mixture is injected into the mold through a nozzle. Due to the low temperature and pressure of the mold, thermodynamic imbalance is rapidly generated, triggering cell nucleation, and cells grow and set in the mold. The ability to use molding dies with specific desired shapes allows for the production of parts with complex geometries. The comparison of extrusion foaming and injection foaming is shown in Table 2.

**Table 2:** Comparison of extrusion foaming and injection foaming.

Comparison Dimension	Extrusion Foaming	Injection Foaming	Ref.
Molding Cycle	Continuous molding, typical cycle < 1 min per unit length (sheet/strand)	Intermittent molding, cycle 3–10 min per part	[64]
Product Structure	Limited to simple shapes (sheet, rod, tube, profile)	Complex shapes	[65]
Cell Orientation	Oriented along the extrusion direction (orientation degree > 0.6)	No obvious orientation (orientation degree < 0.2)	[66]
Production Output	High output, typically >100 kg/h	Low output, typically <20 kg/h	[67]

### 3.2. Optimization of Key Parameters

**Figure 3:**
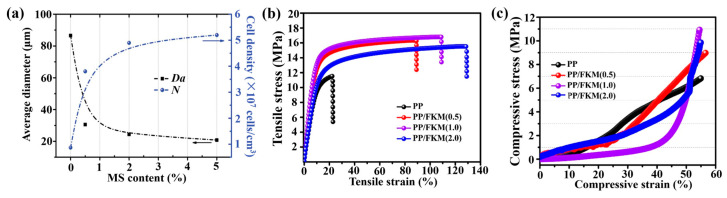
(**a**) Average cell size and cell density of PP and PP/MS nanocomposite foams saturated at 20 MPa and foamed at 154 °C [56]. Mechanical properties of neat PP and PP/FKM foams: (**b**) tensile strength and (**c**) compressive strength [79].

Process parameters exert a significant influence on the stability of the “polymer–gas” homogeneous system and the formation of cellular structures, with the optimization rules of core parameters elaborated as follows:

(1) Regarding the foaming temperature (*T*_f_), the cell density was greater than 10^9^ cells/cm^3^, the expansion ratio was no less than 5, and the cell coalescence rate was less than 5%. It needs to match the crystallization and melting characteristics of PP, typically with an optimal range of 150–170 °C: *T*_f_ < 150 °C will increase the melt viscosity, thereby restricting cell growth; *T*_f_ > 170 °C will cause a sharp decrease in melt strength, easily leading to cell coalescence. Summaries of the properties (average cell size and cell density) of the cellular structure of the PP/MS composite foams as a function of MS content [56] can be seen in Figure 3a.
(2) The optimal saturation pressure (*P*_f_) range is 8–20 MPa, and the cell density increases by more than two orders of magnitude: increased pressure enhances gas solubility in the polymer, promoting heterogeneous nucleation. For the PP/GF30 composite, cell density at *P*_f_ = 16 MPa is 40% higher than that at *P*_f_ = 10 MPa [38]. However, *P*_f_ > 20 MPa triggers excessive scCO_2_-induced polymer plasticization, reducing melt strength and causing cell coalescence [68]. *P*_f_ rises from 10 MPa to 20 MPa, cell density increases by ~2 orders of magnitude, and cell size decreases to <5 μm. This is attributed to stronger nucleation driving force from high-pressure depressurization; meanwhile, enhanced CO_2_ plasticization of the matrix elevates the expansion ratio [69]. The mechanical properties of the resultant PP foam are the important evaluation parameters for potential industrial applications. The tensile stress–strain and compressive stress–strain curves of neat PP and PP/FKM foams are shown in Figure 3b,c.
(3) In terms of the optimization of annealing temperature (*T*_a_), partially melted crystals were formed, with an equilibrium CO_2_ solubility of ≥0.05 g/g. This parameter is mainly used to regulate the crystallization morphology of PP, with an optimal range of 110–130 °C. If *T*_a_ < 120 °C, the CO_2_ solubility will decrease, while *T*_a_ > 130 °C makes it difficult to form partially melted crystals, leading to insufficient melt strength [21]. The broadband dielectric constant and loss of the PP/1.0 wt.% CNT composite before and after the isothermal annealing at different temperatures [70] are measured, which confirms that the annealing treatment under supercritical carbon dioxide can effectively regulate the conductive network and dielectric properties of the composites.
(4) The mold opening distance and rate jointly determine cell expansion space: For the PP/GF30 composite, a 3.4-fold expansion ratio and 75% porosity are achieved at 10 mm distance and 50 mm/s rate; a distance of <2 mm restricts cell growth, while >10 mm induces foam deformation [60]. The density variation in foamed PP and PP/GF30 with mold opening distance [58] is depicted. The cell density/average diameter vs. residence time is demonstrated: extended residence time increases cell density and reduces size, confirming that longer residence time improves the cell structure [71].
(5) For gas selection: scCO_2_ suits most PP composite systems (due to its excellent plasticizing effect), while scN_2_ (with a lower diffusion rate) is preferable for thick-walled component foaming. For thin-walled products (<5 mm), scCO_2_ was preferred; for thick-walled products (>10 mm), scN_2_ was selected. Regarding process integration—it covers three typical processes (precision injection molding, continuous extrusion, batch foaming), with continuous extrusion achieving a 28-fold expansion ratio [72]. Core parameter ranges are clarified, and parameter coupling effects require experiment–model collaborative optimization to stabilize the “polymer–gas” homogeneous system (Table 3).

**Table 3:** Summary of core parameter optimization for supercritical foaming.

Parameter Type	Optimization Range	Influence Mechanism	Refs.
*T*_f_	150–170 °C	<150 °C: high melt viscosity restricts cell growth. >170 °C: sharp drop in melt strength causes cell coalescence.	[73]
*P*_f_	8–20 MPa	Increased pressure enhances gas solubility to promote heterogeneous nucleation. *P*_f_ > 20 MPa: excessive plasticization by scCO_2_ decreases melt strength.	[74]
*T*_a_	110–130 °C	Regulates crystallization morphology. At *T*_a_ = 125 °C, partially melted crystals form to balance melt strength and CO_2_ solubility.	[75,76]
Mold Opening Parameters	Distance 2–10 mm, Rate 50 mm/s	Mold opening distance determines expansion space; if <2 mm it restricts cell growth, if >10 mm it causes deformation.	[77]
Gas Type	scCO_2_, scN_2_	scCO_2_ has strong plasticizing effects. scN_2_ is suitable for foaming thick-walled components.	[78]

## 4. Regulation of Micro-Nano Additives on the Properties of PP Matrix

### 4.1. Regulation of Micro-Nano Reinforcements on the Crystallization Behavior of PP

#### 4.1.1. Improvement of Crystallization Temperature and Rate

The synergy between the micro-nano reinforcement’s heterogeneous nucleation and the partially melted crystals reduces PP nucleation free energy significantly, enabling accelerated crystallization at higher temperatures, while supercritical gas plasticization optimizes crystallization kinetics. For the iPP-20HMSPP system, *T*c increased by 3–5 °C (to 110–123 °C), melting enthalpy and crystallinity rose by 8–10%, which was attributed to HMSPP providing heterogeneous nucleation sites (reducing supercooling) [80]. The PP/30 wt.% glass fiber (GF) system showed Tc elevation by 5–8 °C (113–124 °C), 40% faster crystallization, and a narrowed peak width (~5 °C).

**Figure 4:**
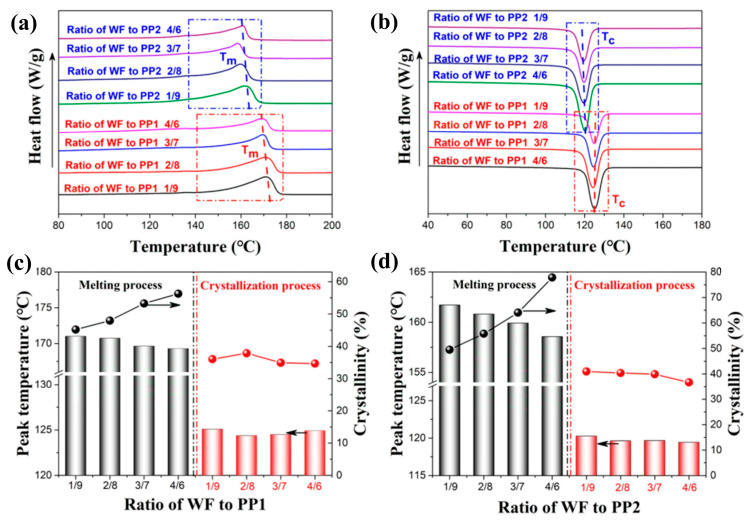
DSC curves (**a**,**b**) and thermal properties (**c**,**d**) of PP1- and PP2-based composites with different WF contents [62].

After modification of the surface hydroxyl groups of glass fiber (GF) with silane coupling agents, van der Waals forces and chemical bonds (Si–O–C bonds) are formed between the GF and polypropylene (PP) molecular chains [58]. Meanwhile, the dipole–dipole interactions between the hydroxyl groups and PP molecular chains further enhance the interfacial adhesion. In supercritical N_2_, carbon fiber (CF) induced PP transcrystallization: *T*c increased by 5 °C (to ~120 °C) versus pure PP (*T*c ≈ 115 °C without N_2_), benefiting from N_2_-reduced chain motion resistance and CF surface nucleation sites [81]. PP/20% CF had 1.5 °C higher *T*c and 20% faster crystallization (the high aspect ratio of CF provided nucleation sites) [81]; PP/30% CF (*T*c ≈ 123.6 °C) showed 30% faster crystallization, with high CF content slightly inhibiting crystallization via chain entanglement but overall improving [77]. Increased sisal fiber (SF) content enhanced *T*c; near-critical length SF formed a lap network for continuous nucleation [47]. Nano-TiO_2_ (specific surface area ≈ 100 m^2^/g) increased *T*c and reduced crystallization activation energy [49]. PBT nanofibers (vs. spherical PBT) better promoted PP nucleation [51]. Nano-carbon black (CB10) raised *T*c by 6 °C and accelerated crystallization [50]. For TPEE/PP blends, Tm increased with PP content (partial compatibility) [82]. The melting and crystallization behaviors of composites with different WF contents were both characterized by differential scanning calorimetry (DSC), and the thermal properties are also shown in Figure 4a–d. According to the listed data, the peak values of DSC curves with the matrix of PP1 were much higher than PP2-based composites with the same WF content. The addition of WF caused heterogeneous nucleation and promoted the perfection of crystal morphology, which led to increased crystallinity and the decreased peak temperatures [62].

#### 4.1.2. Optimization of Crystal Morphology and Size

In polymer matrix composites, micro-nano reinforcements’ steric hindrance effect (inhibiting polymer chain movement, regulating agglomeration and microstructure) originates from their geometry and space-occupying properties, forming physical barriers to adjust intermolecular forces. When synergized with supercritical gas high pressure, it restricts excessive PP spherulite growth; some reinforcements’ interfacial induction regulates crystal growth direction.

PP and PP/CF composite crystallization/melting heat flow: Increasing CF content slightly changes crystallization peaks but shifts melting peaks to higher temperatures, indicating enhanced PP lamellar thickness [77]. PP/25 wt.% LDPE blend nucleation/growth—cocrystallization strengthens spherulite interconnection and mechanical properties [83]. Pure iPP has large, sparsely distributed spherulites with defects, while iPP-20HMSPP exhibits 3–5 times smaller, more uniform spherulites without defects [80]. HMSPP branches restrict disordered chain growth, refining spherulites. CF-induced dendritic crystals show periodic thickness variations at the growth front; edge lamellae migrate and fuse into dense crystals [81].

**Figure 5:**
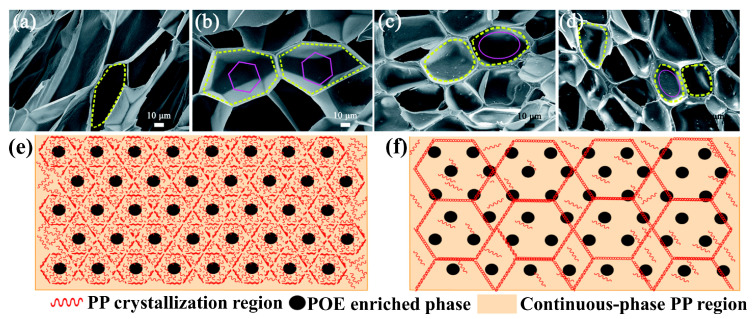
SEM micrographs and cell size distributions of foamed PP (**a**), PP/MS (0.5%) (**b**), PP/MS (2.0%) (**c**), and PP/MS (5.0%) (**d**), saturated at 20 MPa and foamed at 154 °C [56]. Modeling of the crystalline structure of PP/POE blends at 120 °C (**e**) and 137 °C (**f**) [84].

Figure 5a–d show the influence of MS addition on the cell morphologies and cell-size distributions of PP and PP/MS foams. The samples were saturated at 20 MPa and foamed at 154 °C for 10 s. As expected, the pure PP foam presented poor cellular structure and obvious cracked cells. In contrast, PP/MS (0.5%), PP/MS (2.0%), and PP/MS (5.0%) foams exhibited more uniform cell distribution, smaller cell size, a regular polygon cell shape, and well-defined cell walls (Figure 5a–d marked by purple and yellow circles). The cells were strongly in contact by the junction regions of three contacting cells of the different-content PP/MS foams [56]. The relevant crystallization mechanism is illustrated in Figure 5e,f, where the orange part represents the continuous-phase PP region, the black solid part signifies the POE-enriched phase, and the red wavy line part indicates the PP crystallization region [84].

#### 4.1.3. Enhancement of Crystal Structure Stability

Specific micro-nano reinforcements induce stable PP crystals; combined with supercritical gas-enhanced crystal perfection, they improve thermal/melt resistance to adapt to supercritical foaming’s high-temperature environment. Additives stabilize PP’s crystalline structure via chain entanglement/interfacial interactions (maintaining α-crystal, enhancing transcrystallinity, refining grains, guiding orientation).

**Figure 6:**
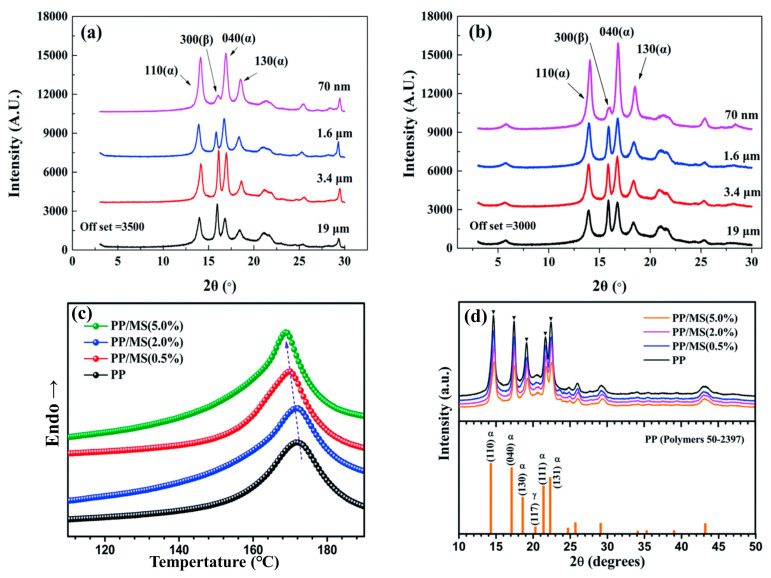
The XRD spectra of composites with different particle sizes of inorganic particles added. (**a**) PP/CaCO_3_, (**b**) PP/MMT [63]. (**c**,**d**) DSC curves and X-ray diffraction patterns of PP and PP/MS samples with different MS contents [56].

All samples exhibit only α-crystal peaks (no β-crystal peaks) with no shift after multiple heating cycles, confirming stable structures [80]. Specifically, HMSPP preserves α-crystal stability via branch-PP chain entanglement; CF optimizes α-crystal content (no transformation) with peak intensity, first increasing, then decreasing (stable position) at 0–20 wt.% CF [81]. The CF/PP interface is smooth, with sparse transcrystals in the first crystallization; after multiple crystallizations, it becomes serrated with 2–3 times higher transcrystalline density (no fragmentation) due to strong CF-PP interfacial interactions [81]. Molding/filler systems affect the crystals: traditional compression molding (CM) causes random PP orientation (diffused α-peaks); pressure-induced flow (SM) + CNTs/CB (1:1) induces flow-direction orientation (distinct arc α-peaks, narrowed width) with 5 wt.% filler being optimal (CNTs-CB network guides chain ordering) [48]. In foaming systems, fiber-added composites show higher crystallinity than pure PP (fibers exert nucleation effects; cellulose restricts amorphous chain movement) [47]. For PP/wood flour/3% TiO_2_, α-peak intensity is 15% higher, FWHM decreases from 0.8° to 0.5°, and grain size is refined by 40% (42 nm → 25 nm); the crystal form is stable after multiple foaming cycles (TiO_2_ restricts chain relaxation) [49]. PP/CB10 maintains α-crystal stability and refines grains via uniform CB dispersion and van der Waals forces with PP [50]. The XRD results of the plastic parts with two reinforcing phases are shown in Figure 6a,b. The characteristic peak of the a-crystal of the (110), (040), and (130) planes appeared at 2*θ* = 14.1°, 16.9°, and 18.5°, respectively, and the characteristic *B*-crystal peak of the (300) plane was located at 2*θ* = 15.9°. The appearance of the *B*-crystal in all plastic parts indicated that both MMT and nano-CaCO_3_ acted as nucleating agents and promoted the formation of the *B*-crystal [63]. Figure 6c presents the DSC melting curves of pure PP and PP/MS composites with different MS loadings (0.5%, 2.0%, 5.0%), showing that the melting peak temperature of the samples gradually decreases with the increase of MS content from 0% to 5.0%. Figure 6d displays the X-ray diffraction patterns of pure PP and PP/MS composites with different MS contents, all of which exhibit the typical diffraction characteristics of α-PP, with the peak positions consistent with the standard PP crystal structure [56].

### 4.2. Regulation of Micro-Nano Reinforcements on the Rheological Properties of PP

#### 4.2.1. Regulation Pathways

Micro-nano reinforcements regulate PP’s rheological properties via the core mechanism of “physical entanglement-three-dimensional (3D) network construction-interfacial synergy”. Combined with supercritical gases’ (scCO_2_, scN_2_) plasticization and high-pressure effects, they directionally optimize PP’s melt elasticity (storage modulus G′), viscosity (complex viscosity η), and tensile resistance.

**Figure 7:**
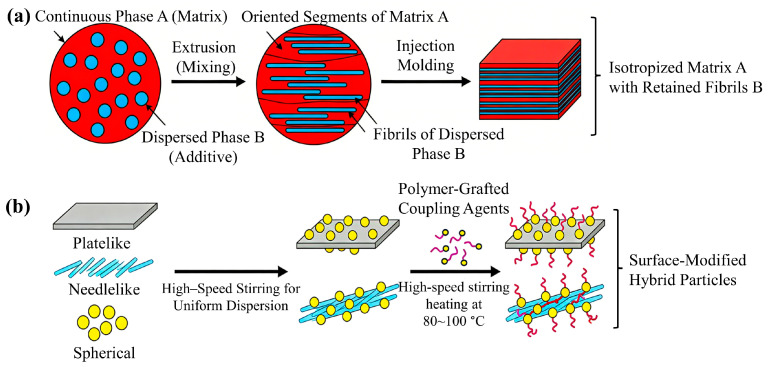
(**a**) Schematic diagram of composite binding. (**b**) Dispersion and function of nano-alumina in the filler system.

Four core regulation pathways exist: (1) Forming continuous 3D networks (via chain entanglement or high aspect ratio) inhibits chain slippage, significantly increasing G′ (Figure 7a). (2) Uniform dispersion enhances internal friction, delaying chain relaxation to improve η (reducing premature gas escape); elastomer-reinforcement synergy balances viscosity and fluidity. (3) Ion-cross-linkable reinforcements form reversible networks, enhancing melt strength while retaining processability. (4) Supercritical gases dually regulate rheology: scCO_2_/scN_2_ plasticization reduces viscosity (aiding gas dissolution), while high pressure promotes chain-reinforcement entanglement.

Melt strength enhanced by partially melted crystals resists cell growth expansion and CO_2_ escape shrinkage, reducing cell defects. Matrix-reinforcement blending undergoes three stages: (1) extrusion “sea–island” morphology, (2) fibrillation-stage highly oriented microfibers, (3) heat-treated isotropic phase A with retained phase B fibers. In situ generated high-aspect-ratio microfibers (from well-dispersed spheres) solve dispersion issues [85]. Microfiber surface-oriented crystals (transcrystalline layers) enhance interfacial adhesion [40] (Figure 7b); dispersed-phase microfibril interfacial debonding under tension connects cells to improve toughness.

#### 4.2.2. Regulation Effects

**Figure 8:**
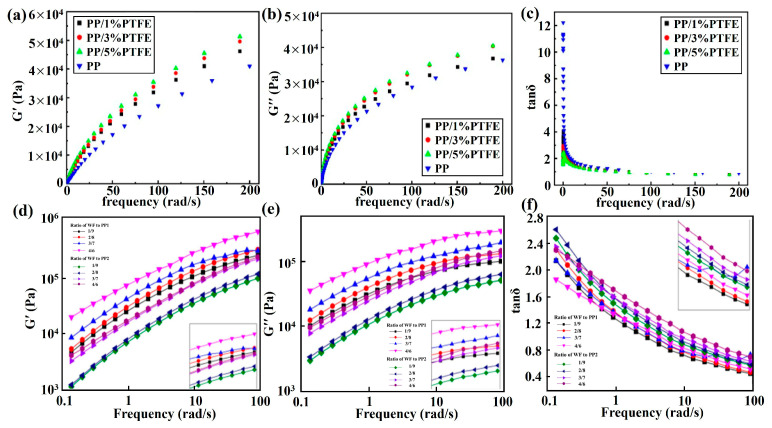
(**a**–**c**) Rheological properties of PP, PP/PTFE and PP/PTFE/MWCNTs (tanδ, G′, G″) [57]. Storage modulus G′ (**d**), loss modulus G″ (**e**), and loss tangent tanδ (**f**) of PP1- and PP2-based composites with different WF contents [62].

For fibrous additives: At 190 °C, 20 wt.% CF increases PP’s G′ (≈10^5^ Pa) by one order and η (≈5 × 10^4^ Pa·s) fivefold vs. pure PP (≈10^4^ Pa G′, ≈10^4^ Pa·s η) [81]. Moreover, 5 wt.% carbon nanofibers both enhance PP’s G′ and η eightfold [82]. Mechanism: Fibers form “fiber–fiber” networks to boost melt elasticity/resistance. For nanoparticles/clay: At 190 °C and 1 Hz, 3 wt.% nano-TiO_2_ increases PP’s η by 50% (≈1.5 × 10^4^ vs. 10^4^ Pa·s) and reduces tanδ (≈0.7) [48]; 3 wt.% nano-clay doubles PP’s G′ (≈2 × 10^4^ Pa) and η (≈2 × 10^4^ Pa·s) via lamellar networks [86,87]. For hybrid fillers: At 180 °C, 5 wt.% CNTs/CB (1:1) enhances PP’s G′ (≈10^4^ Pa) and η (≈10^4^ Pa·s) by two orders of magnitude (“line-point” network) [48]. At 190 °C, PP/3% MWCNT/2% PTFE shows double G′/η vs. PP/3% MWCNT (“tube-line” network) [57], Figure 8a–c. As shown in Figure 8d–f, PP1-based wood–polymer composites showed a higher storage modulus, loss modulus and complex viscosity than PP2-based composites, which were affected by the rheological properties of the matrix [62].

### 4.3. Regulation of Micro-Nano Reinforcements on the Foaming Performance of PP

#### 4.3.1. Improvement of Cell Nucleation Efficiency

The micro-nano reinforcements’ heterogeneous nucleation effect is key to improving PP cell nucleation efficiency—their surface active sites and high specific surface area reduce the nucleation free energy barrier, with the high-pressure dissolution of supercritical gas further enhancing efficiency. Chai et al. [49] found nano-TiO_2_ (≈50 m^2^/g) synergized with wood fiber (WF) to form dense nucleation sites at WF/PP interface. At 3 wt.% nano-TiO_2_, 10 MPa, and 15 min scCO_2_ saturation, WF/PP cell density reached 2.8 × 10^10^ cells/cm^3^ (3-fold higher than without nano-TiO_2_). XRD showed nano-TiO_2_-induced increased α-crystals optimized nucleation site stability. Liu et al. [81] studied CF in 13.79 MPa supercritical N_2_: The CF rough surface grooves and transcrystalline induction formed high-concentration nucleation sites. At 20 wt.% CF, 130 °C, PP/CF nucleation efficiency was 2.7-fold higher than pure PP (1.8 × 10^12^ cells/cm^3^). AFM confirmed that CF-induced dendritic crystals trapped scN_2_ to promote nucleation. Crystallization characteristics affected nucleation: PP and PP/CF had *T*c ≈ 124.6 °C; 20 wt.% CF increased *T*m by 2.4 °C. PP/CF crystallinity was higher than pure PP (56.8%): 5 wt.% CF increased it to 58.17%, and 30 wt.% CF reduced it to 52.17% (chain entanglement) [77]. CF induced preferential nucleation around fibers, shortening crystallization time and increasing grain growth rate.

#### 4.3.2. Regulation of Cell Size and Density

The synergy between micro-nano reinforcements’ steric hindrance and melt elasticity restricts excessive cell growth and optimizes uniformity; combined with supercritical gas pressure regulation, it refines cell size and controls density. Wang et al. [86] found that 3 wt.% nano-clay (interlayer traps scCO_2_, delaying gas diffusion) and 9 wt.% PP-g-MAH (enhances compatibility) reduced the cell size coefficient of variation (CV) to <15% in B-PP extrusion foaming. HMSPP content significantly regulates the iPP cell structure [80]: pure iPP has a low nucleation density (5.07 × 10^5^ cells/cm^3^) and coarse cells (174.63 μm); 20 wt.% HMSPP increased the density to 5.40 × 10^11^ cells/cm^3^ (≈10^6^-fold higher efficiency), refined the size to 2.17 μm, and the expansion ratio to 5.34. The optimal annealing temperature (*T*_a_ = 125 °C) achieved the highest nucleation efficiency with stable performance after multiple cycles. Core mechanisms: (1) The HMSPP long branches induce small α-crystals (heterogeneous nucleation sites) and enhance melt strength (inhibiting coalescence); (2) *T*_a_ modulates crystal perfection, balancing scCO_2_ solubility and melt strength.

Crystallization characteristics significantly affect the PP composite foam cell structure. PP/30 wt.% glass fiber (GF30) exhibited optimal crystallinity (~47%) at heating rates of 5 and 10 °C /min; high heating rates reduced the crystallization temperature due to insufficient cooling time [58]. Liu et al. [77] showed that PP and PP/carbon fiber (CF) composites had a crystallization peak temperature (Tp) of ~124.6 °C; 20 wt.% CF increased melting point (Tm) by 2.4 °C (167.7 °C vs. pure PP’s 165.3 °C) [49]. PP/20% bamboo fiber (BF)/5% composite foam had an expansion ratio of 4.5 with <5% variation after multiple cycles (superior to unmodified systems). Core mechanism: BF-formed networks support cell walls, balancing nucleation and growth for stability.

#### 4.3.3. Enhancement of Cell Structure Stability

**Figure 9:**
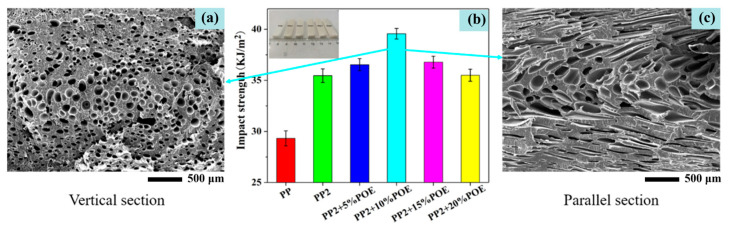
Impact test results: (**a**) cells of vertical section of PP210%POE; (**b**) impact strength; (**c**) cells of parallel section of PP2 + 10% POE [88].

Micro-nano reinforcements can effectively resist cell wall rupture during the expansion of supercritical gases by improving PP’s melt strength (high G′ and strain hardening behavior) and crystal thermal stability. Combined with the high-pressure regulation of supercritical gases, this achieves a balance between high expansion ratio and cell structure stability, avoiding the defects of low expansion ratio and easy collapse in traditional PP foams [46]. Furthermore, Cao et al. [58] prepared 30 wt.% glass fiber (GF)-reinforced PP foams using precision open-mold technology combined with scCO_2_ foaming process; the results showed that the transcrystalline structure induced by GF significantly improved the tensile resistance of the PP melt, with a strain hardening factor of 2.5. When the foaming temperature was 160 °C and the scCO_2_ pressure was 12 MPa, the PP/GF30 foam achieved an expansion ratio of 15 times and a low density of 0.32 g/cm^3^, representing an 87.5% increase compared with that of pure PP foam (expansion ratio of 8 times). Computed tomography scanning results revealed that GF formed a “scaffold” structure on the cell walls, effectively preventing cell collapse, and the foam exhibited a compression rebound rate of 82%. It can be seen from Figure 9 that when 10% POE is used, the foaming effect is better. In pure PP, the foaming effect was poor due to the low melt strength, and many large cells were formed, which easily caused stress concentration and the worst impact toughness [88].

### 4.4. Core Mechanism of Regulation

Micro-nano reinforcements regulate PP crystallization behavior via the core mechanism of “heterogeneous nucleation–interfacial synergy”, which, combined with supercritical gases’ plasticizing and high-pressure effects, directionally optimizes PP crystallization kinetics, crystal morphology, and crystal form stability. Four core pathways are involved:

1. Reinforcements’ surface active sites and high specific surface area adsorb and align PP chains, lowering the nucleation free energy barrier and elevating crystallization temperatures; surface modification enhances interfacial compatibility [16,80].
2. The “melting–annealing–reheating” process forms partially melted crystals that act as physical crosslinks, boosting nucleation site density and restricting chain relaxation [51].
3. Reinforcements’ steric hindrance inhibits spherulite radial growth (refining crystals), synergizing with supercritical high pressure for dual regulation against excessive growth [89].
4. Inducing stable PP crystal forms, coupled with supercritical gas-regulated crystal perfection, improves thermal/melt resistance to support supercritical foaming [77,90].

### 4.5. PP Stability Analysis of Micro Nano Composite Foam

Thermal stability is a critical performance indicator for polypropylene (PP) composite foams in high-temperature service scenarios, and the 5% weight loss temperature (*T*_5_%) obtained via thermogravimetric analysis (TGA) is widely used to evaluate their thermal decomposition behavior under an inert atmosphere. The *T*_5_% values and TGA curve characteristics of PP composite foams are essentially dominated by the type of additives, which can be categorized into four groups—natural fibers, inorganic nanoparticles, polymer microfibers, and elastomers—with distinct influencing mechanisms.

For natural fiber-reinforced PP foams, the *T*_5_% is moderately lower than that of pure PP foam (290~305 °C) [91,92] due to the inferior thermal stability of natural fibers [93,94,95]. Inorganic nanoparticles demonstrate a significant thermal stability enhancement effect on PP foams, primarily relying on their excellent barrier properties and uniform dispersion in the matrix [96,97,98]. As for polymer microfiber-modified PP foams, their thermal stability is determined by the compatibility with the PP matrix and the thermal performance of the microfibers themselves [99,100]. Elastomer-modified PP foams generally show a slight decrease in *T*_5_% compared to pure PP, as elastomers have comparable or lower thermal stability than PP [101,102].

## 5. Application Fields of Supercritical Foamed PP Micro-Nano Composites

### 5.1. Automotive Lightweight Field

Foam performance is closely correlated with its structure—microstructural evolution during foaming endows thermoplastics with diverse functionalities (e.g., thermal insulation, low dielectric constant) [103]. The optimal comprehensive performance relies on targeted design of the key structural/process parameters: cell diameter, density, morphology, expansion ratio, and open cell content [104]. Compared with traditional foaming, microcellular foaming achieves significantly reduced cell diameter and increased density [105,106]; uniform fine cells exhibit excellent mechanical/thermal stability [107], and nanocellular foams outperform microcellular counterparts [108,109].

Based on the cell morphology and open cell content, microcellular foams are categorized into closed-cell, partially open-cell, and open-cell types [110]; reticulated foam (a special open-cell type with 3D reticulated filaments instead of cell walls) [111] is another category. Closed-cell foams dominate lightweight/thermal insulation applications, while open-cell structures are required for sound insulation, oil-water separation, and scaffolds. Controlled cell opening is critical after defining the target functionality and structure.

For structural components, PP/GF30 foam (density 0.32 g/cm^3^, 64.29% weight reduction, flexural strength 58.49 MPa) meets the ISO 178 (Plastics—Determination of flexural properties, the dimensions of the test specimen were 80 mm × 10 mm × 4 mm, with a span length of 50 mm, a loading rate of 2 mm/min, and a test environment temperature of 23 °C) [38]; direct-foamed LFT-D seat frames retain >80% fiber length and enhance specific stiffness by 25% [112]. For anti-collision components, PP/EPDM/BF foam (impact strength 9.62 kJ/m^2^) retains >90% performance at −40–80 °C [39]. PP/wood flour foam (density 0.4 g/cm^3^, formaldehyde emission < 0.1 mg/m^3^) meets GB 18580 (Indoor decorating and refurbishing materials—Limit on formaldehyde emission of wood-based panels and their products) for automotive headliners (the 1 m^3^ climate chamber method was adopted, with a test temperature of 23 °C, relative humidity of 45%, air exchange rate of 1 h^−1^, and test duration of 24 h) [113].

### 5.2. Building Fireproof and Thermal Insulation Field

Micro-nano reinforced PP foam meets the demands for “low thermal conductivity, fire resistance, durability, and self-healing” in building insulation via micro-nano modification (e.g., high-temperature nanofibers, ionic crosslinking, nano-clay doping) and process optimization (scCO_2_ foaming, in situ cooling by adsorbed water).

To meet the requirements of thermal conductivity <35 mW·m^−1^·K^−1^, fire resistance > 150 °C, and long-term energy saving, Zhao et al. prepared PP/PBT nanofiber (diameter < 200 nm) foam with an expansion ratio of 28, a density of 0.032 g/cm^3^, and a thermal conductivity of 32 mW·m^−1^·K^−1^ (outperforming rock wool), reducing building energy consumption by 30% [51]. Li et al.’s EVA/ZnO/PP foam (5 wt.% ZnO) exhibits <5% thermal weight loss at 150 °C, a self-extinguishing time < 10 s, and only a 5% thermal conductivity increase after 12 months of outdoor exposure [114]. Chen et al.’s PP/nano-clay foam, regulated by adsorbed water cooling, achieves a cell size < 20 μm, a thermal conductivity of 29 mW·m^−1^·K^−1^, and <3% volume shrinkage after 100 temperature cycles (−30~80 °C) [115]. In applications, PP/PBT microfiber foam (thermal conductivity 32 mW/(m·K), a service life > 20 years outperforms EPS and rock wool [72], while PP/graphite foam (solar reflectance > 85%) reduces energy consumption by 30% [116].

### 5.3. Packaging and Protection Field

Micro-nano reinforced PP foam achieves functional adaptation in packaging and protection via structural design and process optimization. For precision instruments, PP/carbon nanotube/carbon black foam (volume resistance 10^6^ Ω·cm, average pore size 15 μm) controls electrostatic voltage < 100 V, achieves 88% buffer efficiency, and meets the cleanliness requirements (VOC < 0.1 mg/m^3^) [48]. HMSPP-reinforced iPP foam (density 0.65 g/cm^3^, compression modulus 85 MPa) ensures <0.1% optical accuracy error in 50 cm drop tests and stable performance after damp-heat aging. For transportation equipment, PP/POE foam (PIF-processed, STL 103.56 dB at 1000–6000 Hz, thickness of 5 mm) reduces high-speed rail noise by 15 dB [117], while SF-reinforced PP foam (NRC 0.52, high-frequency sound absorption coefficient > 0.6) lowers aircraft engine noise by 20 dB [118]. In general applications, PP/rubber microfiber foam (1.5 m drop damage rate < 1%) [72], PP/straw fiber foam (degradability >90%, FDA-compliant) [113], PP/nano-SiO_2_ foam (water vapor transmission rate < 1 g/(m^2^·24 h)) [115], PP/graphene foam (surface resistance < 10^6^ Ω) [10,44], PP/GF/CNTs foam, and PP/ceramic microfiber foam (oxygen index > 32%, fire resistance > 90 min) [112] cover diverse needs. PP foam boxes with excellent mechanical properties (withstanding adult stepping) demonstrate their thermal insulation performance (thermal conductivity close to EPPO, stable surface temperature) [119]. Packaging for precision instruments: Priority shall be given to ensuring a cushioning efficiency of ≥85% and electrostatic control with a voltage of <100 V. EMI shielding performance can be reduced to below 20 dB, and CNT content shall be controlled at 5 wt.%. Packaging for electronic devices: Priority shall be given to ensuring an EMI shielding performance of ≥30 dB and volatile organic compound (VOC) control with a concentration of <0.1 mg/m^3^; the cushioning efficiency can be reduced to 75%, and a CNT/activated carbon composite filler shall be adopted.

**Figure 10:**
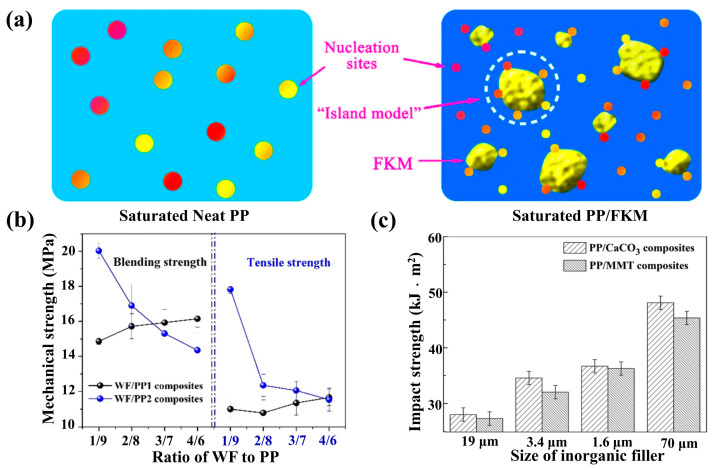
(**a**) Schematic diagram showing the nucleation mechanism of the inner region of neat PP and PP /FKM samples. For clarity, the symbols are not proportional to the real size [79]. (**b**) Blending strength and tensile strength of composites with different resin matrices [62]. (**c**) The impact test results of different composites [63].

As shown in Figure 10a, it is known that foaming is a rapid process in which cells grow in a few seconds, which depends on the thermophysical and rheological properties of PP/CO_2_ mixtures, and this process is related to changes in temperature, pressure, and local stress [79]. The red, orange, and yellow dots in both the neat PP and PP/FKM schematics represent intrinsic heterogeneous nucleation sites with different nucleation activities in the PP matrix, while the large yellow irregular “island-like” structures in the PP/FKM sample are the dispersed FKM phase, which acts as additional high-efficiency nucleation sites at the PP-FKM interface to form the "island model" nucleation mechanism. The mechanical strengths of composites with different matrices were shown in Figure 10b [62]. As the particle size of the inorganic particles was reduced, the impact strength of the composites was remarkably improved [63] (see Figure 10c).

### 5.4. Other Emerging Fields

Micro-nano reinforced PP foams have been applied in electronics/new energy, biomedicine, and environmental protection/emergency response due to their structure-performance advantages.

In electronics/new energy: iPP/CNTs-HDPE/CNTs bilayer foam for 5G casings achieves an EMI SE of 37.32 dB and a 40% weight reduction [44]; PP/graphite foam as a lithium battery insulation pad has a thermal conductivity of 30 mW/(m·K) and >10 min thermal runaway suppression [112]. Wang et al.’s PP/2 wt.% CNT/5 wt.% CB foam exhibits a density of 0.5 g/cm^3^, an EMI SE of 45 dB (1–6 GHz), >85% EMI SE retention after 1000 bends, a compression modulus of 92 MPa, and <30% open cell content (moisture barrier) [48]. Liu et al.’s PP/20 wt.% CF foam (supercritical N_2_ foaming) has a density of 0.35 g/cm^3^, an EMI SE of 32 dB (1–6 GHz), and only a 3 dB EMI SE decrease after 1000 bends [77]. In biomedicine: PP/PCL foam scaffolds for bone repair have a pore size of 100–200 μm, an 85% cell adhesion rate, and 12–24 months degradation [51]; PP/BF foam dressings have an air permeability of >500 g/(m^2^·24h) and >90% antibacterial rate [39]. Bhagat et al.’s PP/near-critical length sisal fiber (SF) foam (145 °C, 100 bar, 15 min scCO_2_ foaming) has >80% open cell content, a compression modulus of 85 MPa (matching soft tissue mechanics), and good biocompatibility (skin wound temporary scaffold) [47]. In environmental protection/emergency response: PP/POE open-cell foam for marine oil spill recovery has a 32-fold oil absorption capacity and 15 reuse cycles [59]. Mi et al.’s PP/mPTFE foam has a superhydrophobic surface (water contact angle > 150°), a 9.1 g/g oil absorption capacity, >90% efficiency retention after five cycles, and good durability [120]. Table 4 compares polymer-based absorbent properties.

**Figure 11:**
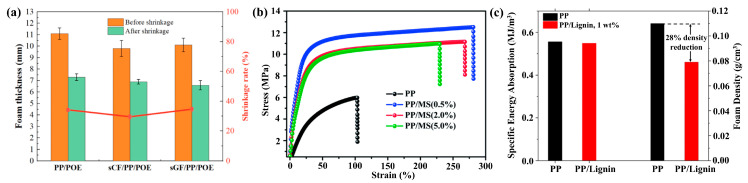
(**a**) Impact of fibers on thermal shrinkage of PP/POE foam [127]. (**b**) Strain–stress curves of foamed pure PP and foamed PP/MS with different MS contents at 154 C and 20 MPa [56]. (**c**) Specific energy absorption (left) and density (right) of the PP and PP/lignin-1% foams [26].

The PP/CF foam effectively shields induced current, exhibiting excellent shielding performance. The thermal conductivity of PP/CF foam on a 100 °C flat plate: the sample temperature increases with CF content, as higher CF content improves thermal conduction paths and enhances in-plane thermal conductivity (beneficial for reducing electronic device heat accumulation) [117]. The electromagnetic shielding mechanism of PP/CF foam is illustrated, including multiple reflection loss, conductivity loss, dipole polarization, and interfacial polarization loss [77]. Despite the improvement in material viscosity and thermal shrinkage to some extent with the addition of carbon fibers in sCF/PP/POE, a further comparison with Figure 11a reveals that its contribution is far less significant than the enhanced thermal shrinkage stability brought about by irradiation crosslinking [127]. The stress–strain curves for the pure PP foam and PP/MS foams prepared at 154 °C and 20 MPa are shown in Figure 11b The stress strength of the PP/MS foam improved significantly from 6.1 MPa to 12.6 MPa, and the tensile strains of the PP/MS foams also increased substantially, reaching 260% [56]. The results are presented in Figure 11c. It can be seen that even though the density of the composite foam is 28% lower than the PP foam, it has almost the same specific energy absorption property, indicating that the PP/lignin foam would allow us to achieve a larger extent of materials and weight savings than its PP counterpart without sacrificing the energy absorption capability of the foam [26].

## 6. Conclusions and Prospects

Notable progress has been made in the theoretical research and application of supercritical foamed PP micro-nano composites, providing eco-friendly solutions for automotive lightweighting, building insulation, and electronic shielding. However, three core bottlenecks restrict industrialization and performance upgrading: raw material compatibility/cost, process stability/scalability, and performance balance/long-term reliability.

At the raw material level: Natural fibers show poor compatibility with non-polar PP (requiring multi-step modification) and agglomerate at high loadings; nano-fillers agglomerate intrinsically, hindering industrial dispersion; high melt strength PP enhances performance but is cost-prohibitive for mass production. In processes: Continuous extrusion foaming fails to maintain “melt–gas” homogeneity (poor consistency); foam 3D printing suffers >30% interlayer bonding loss and low blowing agent retention; batch foaming is controllable but low-efficiency; precision open-mold injection demands high equipment investment. A lack of unified multi-parameter coupling models increases R&D costs. In performance: The inherent strength–toughness trade-off persists; multi-functional integration is challenging; natural fiber moisture absorption and nano-filler migration degrade long-term service performance.

Future research should focus on three breakthrough directions with clear prioritization: (1) High-priority raw material innovation: First, prioritize the development of low-cost, high-performance matrices using recycled PP (directly addressing the cost bottleneck for mass production), followed by interfacial regulation for bio-based/degradable systems and the design of “core–shell” multi-functional reinforcements. (2) Medium-priority process optimization: First, focus on machine learning and online monitoring to build unified process–structure–performance models (reducing R&D costs via multi-parameter coupling), then scale up in situ microfibrillation–continuous foaming equipment and advance foam 3D printing and functionally graded foam technologies. (3) Long-term application expansion: Prioritize deployment in new energy vehicles (aligned with high-demand lightweighting needs), then establish full-life-cycle management (e.g., industrial by-product blowing agents, degradable foams) and formulate the cell structure/long-term performance standards.

In summary, supercritical foamed PP micro-nano composites have broad prospects in high-value fields. Interdisciplinary integration of material science, process engineering, and intelligent technology to address the above challenges will accelerate industrialization, supporting lightweight, energy-saving, and sustainable materials development.

## Figures and Tables

**Table 4:** A comparison of the absorption properties of polymer-based absorbents.

Absorbent Material	Oil Type	Absorption Capacity (g/g)	Form	Ref.
PP/mnPTFE	Chloroform	9.1	Foam	[120]
Polyurethane/MnO_2_ nanowire	Chloroform	49	sponge	[121]
Poly (vinylidene fluoride) (PVDF)	Chloroform	5.58	aerogel	[122]
PVDF/nano-SiC	Engine oil	21.5	foam	[123]
Bagasse	Light oil	3.4	Mesh	[124]
Wood fibers	Crude oil	7	sponge	[125]
Polypropylene	Heavy oil	4.5	Non-woven web	[126]

## Data Availability

No new data were created or analyzed in this study. Data sharing is not applicable to this article.
